# Host Defense via Symbiosis in *Drosophila*

**DOI:** 10.1371/journal.ppat.1003808

**Published:** 2013-12-26

**Authors:** Phineas T. Hamilton, Steve J. Perlman

**Affiliations:** Department of Biology, University of Victoria, Victoria, British Columbia, Canada; University of Wisconsin Medical School, United States of America

## *Drosophila* and Their Defensive Symbionts

**Figure 1:**
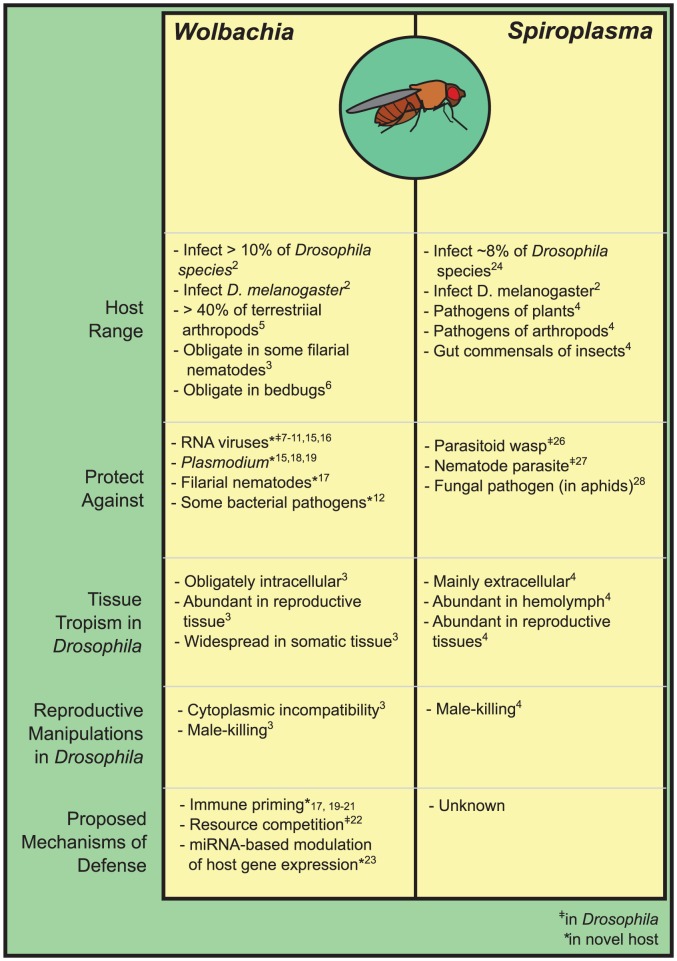
The inherited symbionts of *Drosophila*.

Host-associated microbes have often been studied as pathogens and the causes of disease, but symbiotic microbes that benefit their hosts are now known to be ubiquitous. In particular, insects possess a diversity of bacteria that can defend against natural enemies—*Anopheles* mosquitoes, for example, were recently shown to host a gut bacterium that confers refractoriness to malaria parasites [1]. In *Drosophila*, a key model of infection and immunity, fascinating examples of defense are accumulating, and two lineages of bacteria that infect the genus are now known to be defensive: *Wolbachia* and *Spiroplasma* (Figure 1). Both are vertically transmitted, both are facultative in *Drosophila* in that they are not strictly required by the host, and both infect *Drosophila melanogaster*. Here, we summarize what is known of *Drosophila* as an intriguing and emerging model of defensive symbiosis.

*Drosophila* is an incredibly diverse genus with thousands of species, many of which are infected by *Wolbachia* and *Spiroplasma* [2]. As maternally transmitted symbionts, *Wolbachia* and *Spiroplasma* came to attention in *Drosophila* through their ability to manipulate host reproduction to favor their own transmission. *Wolbachia* are notorious for doing this by inducing cytoplasmic incompatibility (CI), whereby matings between *Wolbachia*-infected males and uninfected females result in the production of few to no offspring [3], providing selective pressure to maintain and rapidly spread *Wolbachia* in host populations. Though *Spiroplasma* are not known to induce CI, both *Spiroplasma* and *Wolbachia* can selfishly distort host sex ratios through male-killing in *Drosophila*, selectively killing the male offspring of infected females [3],[4]. Many strains of *Wolbachia* and *Spiroplasma*, though, do not have such manipulative tendencies, and it has largely been a mystery how they are maintained in host populations. The discovery that they can defend against enemies has gone a long way in explaining their persistence, and has begun to shift our perception of many facultative inherited symbionts from that of manipulative parasites toward helpful mutualists.

## *Wolbachia*: The Master Manipulator

*Wolbachia* are gram-negative α-proteobacteria and are the most widespread and probably best-studied of insect symbionts, infecting upwards of 40% of arthropod species [5]. Most *Wolbachia* are facultative insect symbionts, but the genus is ancient and divergent lineages are obligate symbionts of bedbugs and filarial worms (e.g., [3],[6]). In *Drosophila*, screens have uncovered *Wolbachia* in 8–12% of species, although this probably underrepresents the true frequency of infection [2].

Defensive properties of *Wolbachia* were discovered when screens of *D. melanogaster* for resistance to RNA viruses uncovered an association between resistance and *Wolbachia* infection, with *Wolbachia* inhibiting viral replication and decreasing virus-induced mortality [7],[8]. Strains of *Wolbachia* have since been shown to defend *D. melanogaster*, *D. simulans*, and *D. innubila* against multiple RNA viruses [7]–[11]. As yet, though, *Wolbachia* are not known to defend against other enemies of *Drosophila*, with a lack of defense demonstrated against DNA viruses [7], bacterial pathogens [12],[13], and parasitoid wasps [14]. It remains possible however, that *Wolbachia* may defend against some of these parasites or pathogens in the wild, as laboratory challenges do not always use pathogens that naturally infect *Drosophila* (e.g., the DNA virus used in [7]).

## Spreading the Love: Transforming Disease Vectors with *Wolbachia*

In contrast to *Wolbachia*'s limited defensive effects in *Drosophila*, strains introduced from *Drosophila* into *Aedes* and *Anopheles* mosquitoes (“heterologous” infections) more broadly inhibit the development of diverse parasites and pathogens. These include dengue virus, chikungunya virus, *Plasmodium* spp., and filarial nematodes [15]–[19]—suggesting the possibility of transforming vectors to limit transmission of human disease. *Wolbachia* also causes CI in these novel hosts, providing a ready mechanism for the drive of *Wolbachia* and defensive traits into naïve vector populations. This possibility has spurred interest in the mechanistic basis of *Wolbachia*'s defense, but has also led to a focus on heterologous infections in mosquitoes rather than native infections in *Drosophila*.

In these novel hosts, it is commonly observed that *Wolbachia* causes a barrage of immunological effects that are apparently responsible for defense: these include the induction of the Toll and other host immune pathways, and the production of toxic reactive oxygen species (e.g., [15],[18]–[20]), and are typically accompanied by substantial cost to the host (i.e., *Wolbachia* infections are virulent) (e.g., [19]). Conversely, such effects are absent or attenuated in native *Drosophila* infections that are also defensive [21], raising the question of whether the same defensive mechanisms underlie *Wolbachia*'s effects in native and novel hosts, and whether the dramatic immune induction observed in novel mosquito hosts is the cause of defense, a corollary of a new and virulent *Wolbachia* infection, or both—but as yet it is essentially completely unknown how *Drosophila* are defended. Recently though, *Wolbachia* competition with RNA viruses for cholesterol has been argued to contribute to defense in *D. melanogaster* [22], while *Wolbachia* modulation of a host miRNA to regulate a methyltransferase appears to contribute to viral defense in *Aedes* [23]. Studies have also observed that levels of defense are related to *Wolbachia* density—for example, the protection conferred by *Wolbachia* that naturally infect *Drosophila simulans* is limited to strains that achieve higher densities within the host [9], suggesting that competitive effects or tissue tropism (e.g., overlap in symbiont and parasite infection in host tissues) may be important. Further work is clearly needed to untangle the relative contributions of divergent mechanisms to defense, both in *Drosophila* and in novel hosts that we might seek to transform.

Based on findings of defense and the recapitulation of CI in mosquitoes, researchers have engineered the mass release of *Wolbachia*-infected *Aedes* mosquitoes in Australia to limit the transmission of dengue, and observed the rapid spread of *Wolbachia* through CI [24], serving as a proof-of-concept of vector transformation with *Wolbachia*. While exciting, it remains to be seen how the incidence of dengue in humans will be affected in the study area.

*Wolbachia* have also been introduced into the anopheline vectors of malaria, which they do not naturally infect, and can substantially inhibit the development of *Plasmodium falciparum* in *Anopheles gambiae* [17]. However, efforts at using *Wolbachia* in the biocontrol of malaria have been hampered by difficulty in establishing infections that are efficiently vertically transmitted. Intriguingly though, a *Wolbachia* strain native to *Aedes albopictus* has recently been introduced into *Anopheles stephensii*; in this host it inhibits *Plasmodium* development, is vertically transmitted with high fidelity, and induces CI, suggesting that biocontrol methods using *Wolbachia* in *Anopheles* could be on the horizon [18]. Still, this infection is highly virulent, substantially decreasing the hatch rate of host eggs [18], and whether CI traits are strong enough to offset such costs to maintain *Wolbachia* in this host in the long term is unclear.

## *Spiroplasma*: Under the Radar

*Spiroplasma* are cell-wall-less gram-positive bacteria of the class Mollicutes, and are known as plant and arthropod pathogens, and gut commensals and inherited symbionts in insects [3]. In *Drosophila*, *Spiroplasma* are primarily known as male-killers and are widespread, infecting ∼8% of screened species [25].

Different *Spiroplasma* strains have now been shown to be defensive in two *Drosophila* species [26],[27], as well as in aphids [28], in what appear to be the first examples of *Spiroplasma* behaving mutualistically. In *Drosophila hydei*, *Spiroplasma* increases the survival rate of flies attacked by a parasitoid wasp [26]. In *Drosophila neotestacea*, *Spiroplasma* decreases the size and transmission of a common and virulent nematode parasite of the fly, and restores the fertility of nematode-parasitized flies that are normally sterilized by infection [27]. This strong protective effect has lead to *Spiroplasma*'s rapid continent-wide spread through North American *D. neotestacea* in recent decades [29]. This *Spiroplasma* strain causes no reproductive manipulation, apparently relying solely on the selective advantage of the defense it confers to spread, providing one of the more compelling examples of the importance of defensive symbioses in the wild.

Little is known of the mechanisms by which *Spiroplasma* provide defense, but *Spiroplasma* are phylogenetically distant from *Wolbachia*, underscoring many differences in their biology. *Spiroplasma* typically occur extracellularly in the host hemolymph while *Wolbachia* are predominately intracellular, and *Spiroplasma* cause little apparent immune activation in their hosts [30],[31]. *Spiroplasma* may be more susceptible to immune effectors active in the host hemolymph due to their extracellular lifestyle, and thus under selection to avoid or even suppress host immune activation (e.g., [30]). Mechanisms other than host immune priming might therefore account for *Spiroplasma*-mediated defense. In other defensive symbioses, symbiont-encoded or associated toxins have been implicated [32], and it remains possible that toxins are involved in *Spiroplasma* defensive symbioses.

A recent study also found that *D. melanogaster* infection by a male-killing *Spiroplasma* actually increased mortality from infection by a gram-negative pathogen, while not affecting host survival after challenge by gram-positive or fungal pathogens, demonstrating the complexity and contingency of interactions between hosts, symbionts, and enemies in heritable symbioses [32]. The co-occurrence of *Wolbachia* and *Spiroplasma* in the same *Drosophila* species, and even in the same individuals, provides a powerful opportunity for comparative study of the factors underlying apparently independently evolved defensive symbioses in order to untangle some of this complexity.

## Conclusions and Perspectives

The recent surge of interest in defensive symbioses of insects has roots in our growing awareness of the importance of host microbiomes to health and disease, and in our desire to control disease through engineering the frequency or defensive characteristics of insect symbionts. Despite our yet-limited understanding of specific mechanisms underlying defense, the goal of using symbionts as biological control agents points to unresolved questions regarding the nature of defensive symbiosis, typified by *Wolbachia*'s incongruent effects in heterologous and native infections.

In novel mosquito hosts, upregulation of immune pathways by *Wolbachia* appears to entail increased resistance to diverse infectious agents. In native *Drosophila* hosts, defense appears specific and finely tuned by natural selection, possibly conferring a selective advantage that maintains symbiont infection in host lineages. Understanding whether these observations comprise qualitatively different means of defense or are only superficially divergent and rely on the same underlying mechanisms will be necessary. It also raises the question of the specificity of defense, both in different hosts and against different enemies. Mechanism will also have consequences for evolutionary stability; for instance, symbionts that achieve defense through immune priming with high collateral cost to the host will place strong selective pressure not only natural enemies, but also on the host and symbiont to suppress defensive traits.

If defense does in fact turn out to be a relatively nonspecific consequence of symbiont infection, it raises the question: what infection isn't defensive? Interactions between coinfecting parasites and pathogens abound, some positive, some negative (e.g., [33]). Defining any infection with a negative effect on a coinfection as defensive may not ultimately reflect relationships well. Nonetheless, symbiont-mediated defense in *Drosophila* can be both adaptive and ecologically relevant [26]. Deciding if a symbiont should be considered defensive will require integrating an understanding of the strength of symbiont-mediated protection and costs of symbiont infection with the frequency and effects of parasites and pathogens in the wild. Unfortunately, such data are often lacking.

Other microbes may also be important. In bumblebees, gut microbes can protect against trypanosomatid parasites [34], and, as mentioned, a gut bacterium of *Anopheles* provides refractoriness to *Plasmodium* [1]. That such defensive effects result from members of the *Drosophila* microbiome seems probable, but their study is only in its infancy. Still, in the past decade, great strides have been made in understanding the importance, evolutionary consequences, and possible mechanisms of defensive symbioses. We hope that further work in *Drosophila* will continue to drive this trend.
